# A Novel Type of Ureteral Stents in the Treatment of a Bilateral Iatrogenic Transaction of the Ureters

**DOI:** 10.1155/2013/302351

**Published:** 2013-07-29

**Authors:** Ernesto Mazza, Francesco Mondaini, Dalmar Abdulcadir, Claudio Raspanti, Michele Citone, Alberto Lapini

**Affiliations:** ^1^Department of Extravascular Interventional Radiology, Careggi University Hospital of Florence, Largo Brambilla 3, 50100 Florence, Italy; ^2^Department of Radiology, Careggi University Hospital of Florence, Largo Brambilla 3, 50100 Florence, Italy; ^3^Department of Radiology, New Hospital of Mugello, Viale della Resistenza 60, Borgo San Lorenzo, 50032 Florence, Italy; ^4^Department of Urology 1, Careggi University Hospital of Florence, Largo Brambilla 3, 50100 Florence, Italy

## Abstract

This report illustrates the case of a patient who suffered an iatrogenic complete injury of both ureters after a complex surgical procedure to remove a large sacral chordoma. Ureteral recanalization was achieved with two removable, autoexpandable, and polytetrafluoroethylene covered nitinol stents. To our knowledge, we describe the first application of this type of stents to treat a bilateral ureteral transection. Despite the bad general conditions of the patient, the ureteral stents successfully restored and maintained the bilateral ureteral continuity.

## 1. Introduction

Ureteral stents have been used since 1978 when the double-J stent and the single pig-tail stent were invented by Finnley and Hepperlen [1, 2] and are now part of the “armamentarium” of the urologists and interventional radiologists.

Over the years, many changes were made in the ureteral stent design, material composition, and stent coating in order to maintain the ureteral continuity and to avoid complications.

The Niti-S ureteral stent is a novel kind of polytetrafluoroethylene (PTFE) covered, removable stent available for the treatment of ureteral stenosis.

This report presents the case of a patient who suffered a complete bilateral iatrogenic ureteral lesion after a complex surgical procedure to remove a large sacral chordoma. Ureteral recanalization was achieved with two removable, autoexpandable, and covered nitinol stents.

It was possible to completely restore the bilateral ureteral continuity with a combined anterograde and retrograde approach and the positioning of a novel type of removable autoexpandable nitinol covered stent (Niti-S Uventa, by Taewoong Medical Co., Ltd., Republic of Korea).

## 2. Case Presentation

**Figure 1:**
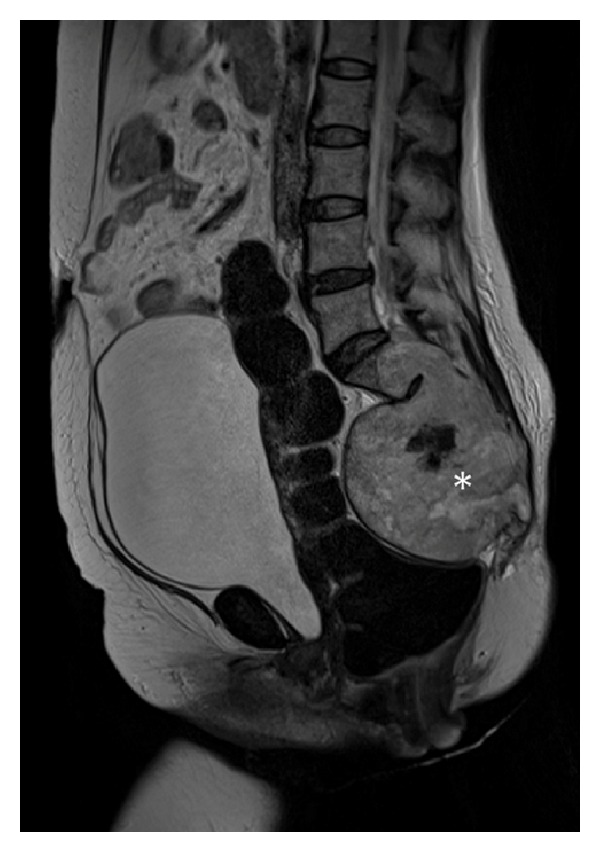
MRI scan of the large chordoma (white star) of the sacrum: T2-weighted sagittal image.

A 56-year-old woman was admitted in March 2011 to the orthopaedic oncology unit for a large sacral chordoma of 15 × 11 × 9 centimetres extending from L5 to the coccyx, infiltrating the rectum, sigmoid colon, sacroiliac joints, piriformis muscle, gluteus medius muscle, and both ureters (Figure 1).

In June 2011, the patient was treated with a radical sacrectomy, the resection of L5, and a left hemicolectomy. Two double-J stents were then cystoscopically positioned in both ureters.

Soon after surgery, the patient suffered a conspicuous loss (1000 mL/die) of urine from the surgical wound due to a bilateral ureteral transection.

**Figure 2:**
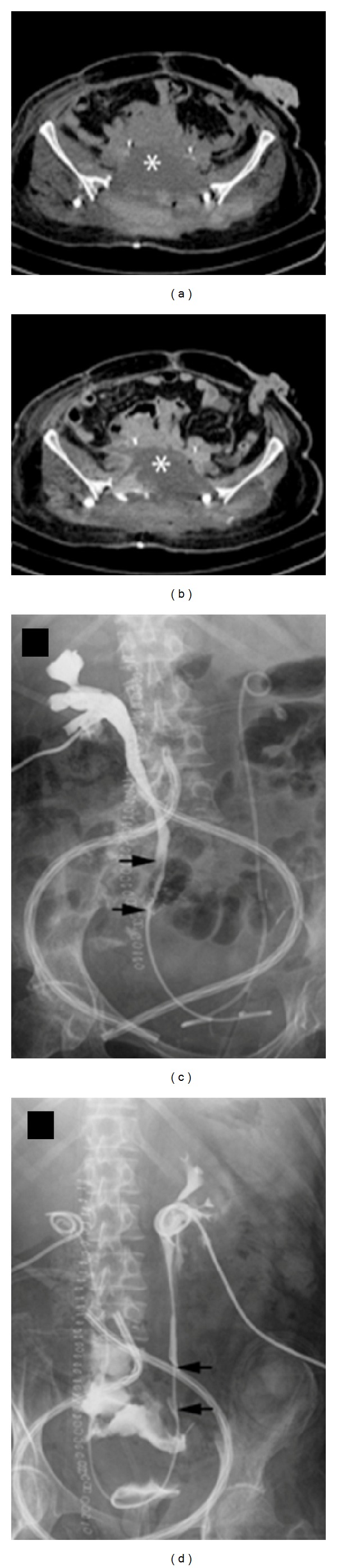
Noncontrast-enhanced (a) and contrast-enhanced (b) CT scans of the pelvis after radical sacrectomy. A large fluid collection (white star) surrounds the ureters where two double-J stents were positioned. Pyelography through the bilateral nephrostomies showing the transection of both ureters (black arrows) and contrast media leakage ((c) and (d)).

A contrast-enhanced CT scan showed a large urinoma extending from L4 to the pelvis and a contrast media leakage from the distal part of both ureters (Figures 2(a) and 2(b)). The recovery of the surgical lesion was impaired by the urine loss in the pelvis; therefore, a bilateral nephrostomy was performed (Figures 2(c) and 2(d)).

General conditions of the patient deteriorated and she became septic; in addition, the surgical wound did not show any sign of healing, despite the nephrostomies. Two vascular plugs were then cystoscopically positioned inside the middle ureters in order to deviate the urine flow towards the nephrostomies. After a temporary reduction of the leakage volume (from 1000 to 200 mL/day), the urine volume inside the pelvis increased again.

Considering the performance status of the patient, a surgical approach would have been too invasive and invalidating for the patient. It was then decided to use two covered, removable, and self-expandable nitinol stents to restore the ureteral continuity. It was planned to leave the stents permanently in the ureters.

**Figure 3:**
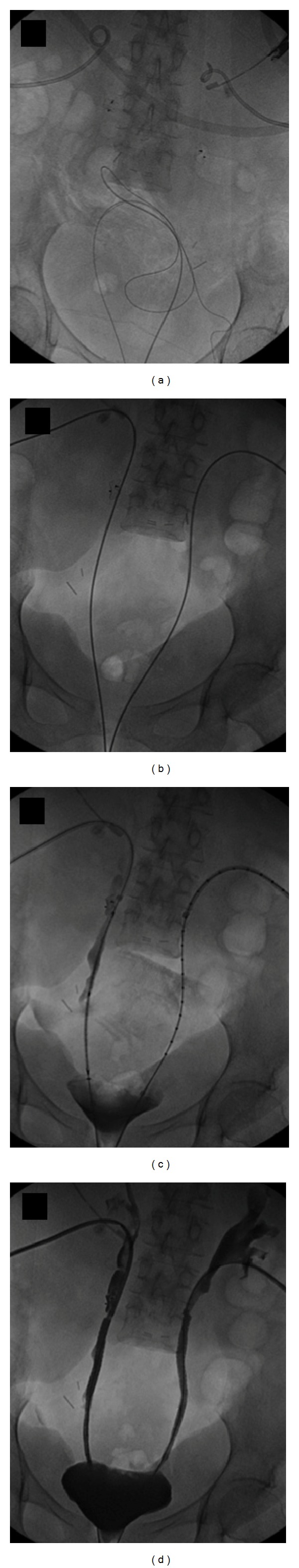
Rendez-vous procedure for installing the PTFE nitinol stents.

Using a “rendez-vous” technique, the guide-wires introduced in the ureters anterogradely and retrogradely met and the vascular plug that had been previously positioned in the right ureter was removed. It was not possible to remove the vascular plug in the left ureter. Subsequently, the two nitinol stents were positioned in the ureters covering the wall lesions on both sides. The final check showed a normal flow of the contrast media through both ureters to the bladder without any leakage in the pelvis (Figure 3).

**Figure 4:**
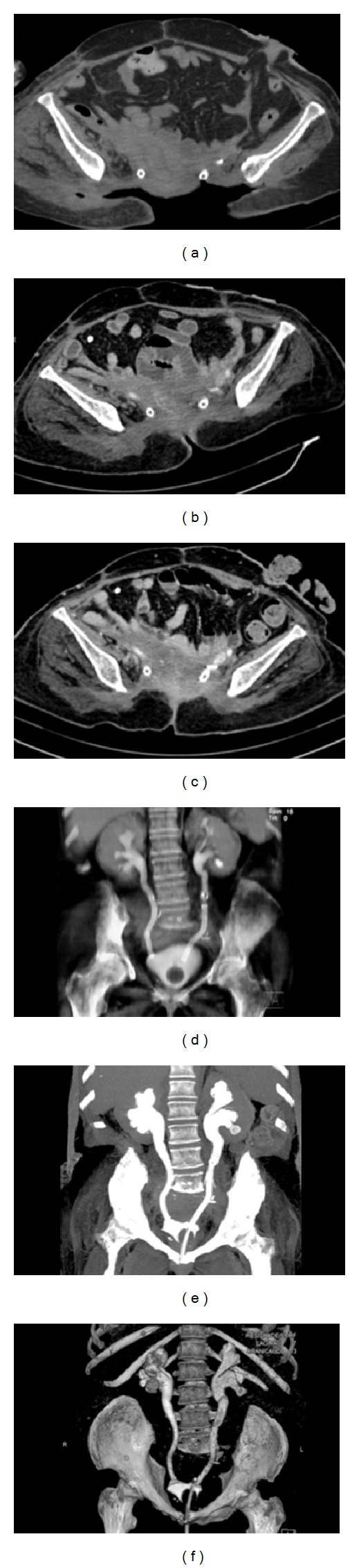
Contrast-enhanced CT scan (a) the day after the ureteral stents were positioned. Contrast-enhanced CT scan performed in January 2012 (b) and in April 2012 (c): the surgical wound shows a progressive healing and there is no ureteral leakage. Maximum intensity projection (MIP) CT-reconstruction showing the correct position of the two ureteral stents after 8 (d) and 18 months from the procedure (e). 3D CT-volume rendering after 18 months from the procedure (f).

The follow-up contrast-enhanced CT scans performed every 3 months did not show any leakage of contrast media, whereas there was a progressive healing of the surgical wound (Figures 4(a)–4(d)).

Last contrast-enhanced CT scan performed 18 months after treatment did not show any leakage of urine while the patency of both ureters was still fully preserved (Figures 4(e) and 4(f)). 

## 3. Discussion

Installing a ureteral stent is nowadays a routine interventional procedure and has replaced the surgical approach, having a significantly lower morbidity and mortality rates [3].

Percutaneous nephrostomy corrects the abnormality for a short period of time, but it has an important negative impact on the quality of life of the patient; plus it is associated with important complications such as infection and haemorrhage.

The conventional double-J stenting technique is routinely used even for long-term treatments and is much more accepted by the patients than percutaneous nephrostomy. On the other hand, double-J stents need to be changed every 3–6 months [3] and they have frequent complications such as migration, encrustation, fracture, edema, and fibrosis [4]. Moreover, the double-J stents usually are rarely sufficient in controlling the leakage of urine in cases of transection of the ureters.

New stent designs and materials have been developed in the last few years, such as the metallic, covered and noncovered, expandable, and permanent stents used in the treatment of a variety of diseases, principally of the vascular and biliary systems.

Although some authors [5] have initially reported good results of the noncovered stents, in the last few years some articles [6, 7] reported low rates of primary ureteral patency after the positioning of noncovered ureteral stents due to a marked mucosal hyperplastic reaction through the mesh of the stent itself.

Covered stents have not shown good results either in restoring or maintaining ureteral patency [8]; moreover, they showed a high rate of migration [9]. 

Restoring the ureteral continuity in patients with comorbidities and low performance status still constitutes a therapeutic dilemma.

This report shows the use of a novel type of stent that demonstrated to be very effective in maintaining a long-term ureteral patency in a patient with a high surgical risk. 

The Niti-S ureteral stent (Uventa, by Taewoong Medical Co., Ltd., Republic of Korea) is an implantable, self-expandable, covered, and metallic stent. It is constructed with three layers: the inner and the external ones are made of nitinol and the middle one is made of a biocompatible PTFE membrane that prevents the risk of tumour ingrowth and reduces encrustation.

When the stent is correctly installed, it shows a high radial force so that it can be used to treat intrinsic and extrinsic neoplastic stenosis (ureteral metastasis, encrustation of double-J stents, intrinsic/extrinsic compression due to malignancies, direct malignant invasion of the ureters, and stenosis due to fibrosis).

Chung et al. [9] have compared the Niti-S covered stent to a noncovered stent in canine ureters and have reported positive results in maintaining ureteral patency compared to the near-total occlusion of noncovered stents. Furthermore, the authors reported that stent migration did not occur.

Arguiñarena and del Busto have reported a low rate of calcification and nonobstructing mucosal hyperplasia using PTFE covered nitinol stents in treating various cases of ureteral stenosis; moreover, they assessed that the ureteral patency was maintained in all cases [10].

In conclusion, PTFE covered nitinol stents were shown to be effective in the treatment of complete bilateral ureteral lesions and a safe alternative to invasive surgical procedures.
